# MYSM1 induces apoptosis and sensitizes TNBC cells to cisplatin via RSK3–phospho-BAD pathway

**DOI:** 10.1038/s41420-022-00881-1

**Published:** 2022-02-26

**Authors:** Xiaolin Guan, Xin Meng, Keyu Zhu, Jinyan Kai, Yixuan Liu, Qian Ma, Ying Tong, Hui Zheng, Suhong Xie, Xiaolu Ma, Yanchun Wang, Renquan Lu, Lin Guo

**Affiliations:** 1 Department of Clinical Laboratory, Fudan University Shanghai Cancer Center, Shanghai, China; 2 Department of Oncology, Shanghai Medical College, Fudan University, Shanghai, China

**Keywords:** Chemotherapy, Breast cancer

## Abstract

Breast cancer is one of the leading causes of mortality among women. Triple-negative breast cancer (TNBC) is responsible for a large percentage of all breast cancer deaths in women. This study demonstrated the function of Myb-like, SWIRM, and MPN domains 1 (MYSM1), an H2A deubiquitinase (DUB), in TNBC. MYSM1 expression was drastically decreased in breast cancer, especially in TNBC, suggesting a potential anticancer effect. Overexpressing and suppressing MYSM1 expression in TNBC cell lines led to significant biological changes in cell proliferation. Furthermore, MYSM1 overexpression increased cisplatin-induced apoptosis, which might be attributed to RSK3 inactivation and the subsequently decreased phosphorylation of Bcl-2 antagonist of cell death (BAD) (Ser 112). The findings suggest that MYSM1 is a potential target for regulating cell apoptosis and suppressing resistance to cisplatin in TNBC.

## Introduction

Breast cancer is the most common malignant tumor and the prominent cause of death in women. Triple-negative breast cancer (TNBC) is the most dangerous subtype with the worst therapeutic prognosis and outcomes due to the absence of receptors for estrogen, progesterone, and HER2. Chemotherapy is the standard treatment for TNBC, and cisplatin is widely used in the clinical management of TNBC [1–3]. Therefore, the loss of sensitivity to chemotherapy is the primary cause of therapeutic failure.

Histone ubiquitination is considered the most common epigenetic modification underlying gene transcriptional regulation, apoptosis, cell cycle, and DNA damage repair [4]. Myb-like, SWIRM, and MPN domains 1 (MYSM1) is a specific deubiquitinase (DUB) of histone H2A. MYSM1 regulates transcription by coordinating histone acetylation and deubiquitination while disrupting the histone H1 association between nucleosomes [5].

MYSM1’s biological functions have been primarily investigated in hematopoietic and immune systems. The role of MYSM1 in solid tumors is rarely reported. Zhu et al. [5] demonstrated that MYSM1 was involved in the transcriptional regulation of androgen receptor (AR)-dependent gene activation, whereas uH2A level in prostate cancer decreased significantly and might represent a tumor marker. Sun et al. [6] reported that MYSM1 downregulation promoted cell proliferation and inhibited cell aging in castration-resistant prostate cancer (CRPC). MYSM1 is also associated with adverse outcomes in colorectal cancer [7] and melanoma [8], indicating a potential tumor suppressor role in cancer.

This study demonstrates that MYSM1 is required for sensitizing TNBC cells to cisplatin by enhancing cell apoptosis. The fundamental mechanism entails the depletion of RSK3, which blocks the phosphorylation of Bcl-2 antagonist of cell death (BAD) and activates the BAD-mediated cell death. These findings indicate a potential role of MYSM1 in maintaining sensitivity to TNBC chemotherapy.

## Results

### MYSM1 is poorly expressed in TNBC

**Fig. 1:**
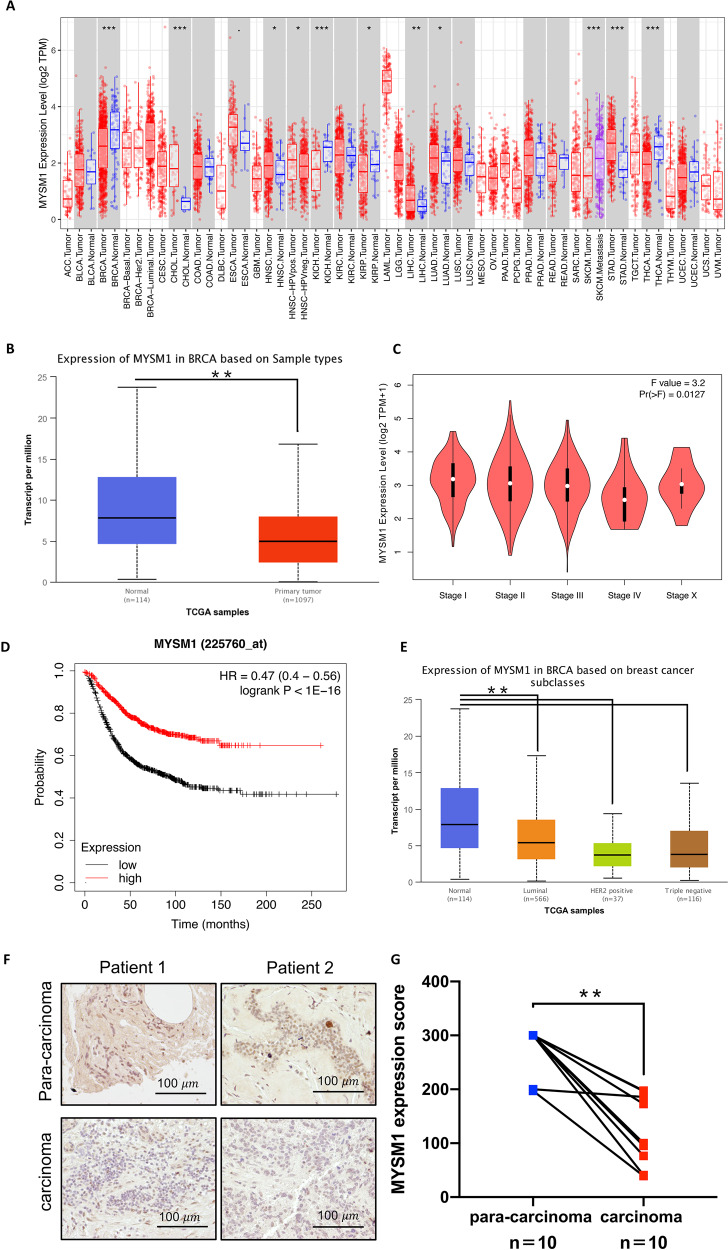
Low expression of MYSM1 is correlated with a poor progression in breast cancer. **A** MYSM1 expression levels in different types of cancers. The statistical significance was determined via the Wilcoxon test (annotated by *P < 0.05; **P < 0.01; ***P < 0.001). Data were acquired from the TCGA database and analyzed using TIMER bioinformatics. **B** MYSM1 mRNA levels in breast normal tissues and primary breast tumor tissues; data were acquired from the TCGA database and analyzed via UALCAN bioinformatics. **C** One-way ANOVA was used to analyze MYSM1 expressions based on the primary pathological stage as a variable. Data were acquired from the TCGA databases and analyzed via GEPIA bioinformatics. **D** The relapse-free survival curve of groups of patients with high and low levels of MYSM1. Data were acquired from the Kaplan–Meier plotter. **E** MYSM1 levels in breast cancer subclasses based on the TCGA database and analyzed via UALCAN bioinformatics. **F** The representative immunohistochemical images of MYSM1 protein levels in carcinoma and para-carcinoma tissues of patients with TNBC. Scale bars: 100 μm. **G** Quantification of MYSM1 expression in carcinoma and para-carcinoma tissues of patients with TNBC using the Image-Pro Plus software. *P < 0.05 and **P < 0.01.

The analysis of the TCGA database by TIMER bioinformatics [9, 10] revealed varying levels of MYSM1 in normal and tumor tissues (Fig. 1A). MYSM1 was found to be drastically downregulated in breast cancer [11] (Fig. 1A, B). MYSM1 levels were similarly reduced at advanced pathological stages (Fig. 1C). The relapse-free survival curve obtained from the Kaplan–Meier plotter [12] suggested that the increased expression of MYSM1 was linked to a possible improved prognosis (Fig. 1D). Furthermore, MYSM1 levels were remarkably lower in HER2-positive breast cancer and TNBC than in the luminal type, indicating that MYSM1 might have specific functions in HER2-positive breast cancer and TNBC (Fig. 1E). TNBC was chosen as the focus of this study because it had the worst outcomes and prognosis among all breast cancer subtypes. The immunohistochemical analysis of ten pairs of TNBC para-carcinoma and TNBC carcinoma tissues was performed to validate MYSM1 expression. TNBC carcinoma tissues showed a distinct MYSM1 downregulation compared to paired para-carcinoma tissues (Fig. 1G). Representative images depicted these findings (Fig. 1F).

These data confirmed the low expression of MYSM1 in TNBC and its potential anticancer effect. Furthermore, they established the foundation for the rest of this research to explore the biological effects of MYSM1 in TNBC.

### MYSM1 suppresses cell proliferation, and cisplatin reinforces the suppressive effect

**Fig. 2:**
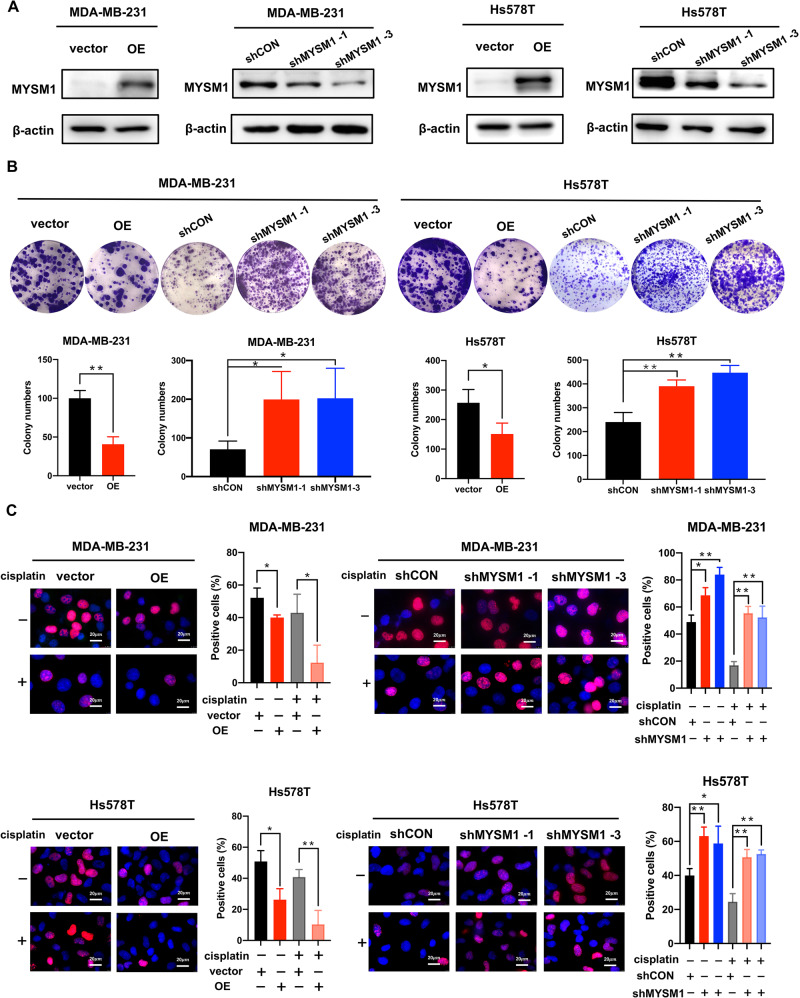
MYSM1 suppresses cell proliferation, and the suppressive effect is reinforced by cisplatin treatment. **A** MYSM1 protein levels in MDA-MB-231 and Hs578T cells with stable MYSM1 overexpression and knockdown and their corresponding negative control cells were detected by western blot. **B** Colony formation assay was performed in MDA-MB-231 and Hs578T cells with stable MYSM1 overexpression and knockdown and their corresponding negative control cells. **C** MDA-MB-231 and Hs578T cells with MYSM1 overexpression and knockdown, and their corresponding negative control cells were treated with or without cisplatin (5 µg/mL) for 48 h, followed by the determination of cell proliferation via EdU incorporation. Scale bars: 20 μm. All data in the graph bars represent means ± SD. *P < 0.05 and **P < 0.01.

MDA-MB-231 and Hs578T cell lines with stable MYSM1 overexpression and knockdown were established via lentivirus infection to investigate the specific biological functions of MYSM1 in TNBC. Western blot was used to determine the efficiencies of overexpression and knockdown. MYSM1 protein levels were much lower in TNBC cells transfected with two distinct shRNAs (shMYSM1) than in the control (shCON) group (Fig. 2A). MYSM1 levels were likewise significantly higher in TNBC cells that overexpressed MYSM1 than in the empty vector (vector)-transfected cells (Fig. 2A). The colony formation assay revealed that MYSM1 overexpression decreased the colony formation, whereas the knockdown of MYSM1 had the opposite effect (Fig. 2B).

MYSM1 significantly inhibited cell proliferation. EdU incorporation attenuated in cells overexpressing MYSM1; the opposite trend was observed in cells with MYSM1 knockdown (Fig. 2C). However, the treatment with cisplatin (5 µg/mL) for 48 h altered the EdU incorporation range even more (Fig. 2C), meaning that cisplatin increased the proliferation effect caused by MYSM1. These results suggested a potential role of MYSM1 in cisplatin treatment.

These results indicated that MYSM1 suppressed cell proliferation and might play an essential role in mediating the biological effects of cisplatin.

### MYSM1 increases apoptosis and ROS production and decreases cell viability upon cisplatin treatment

**Fig. 3:**
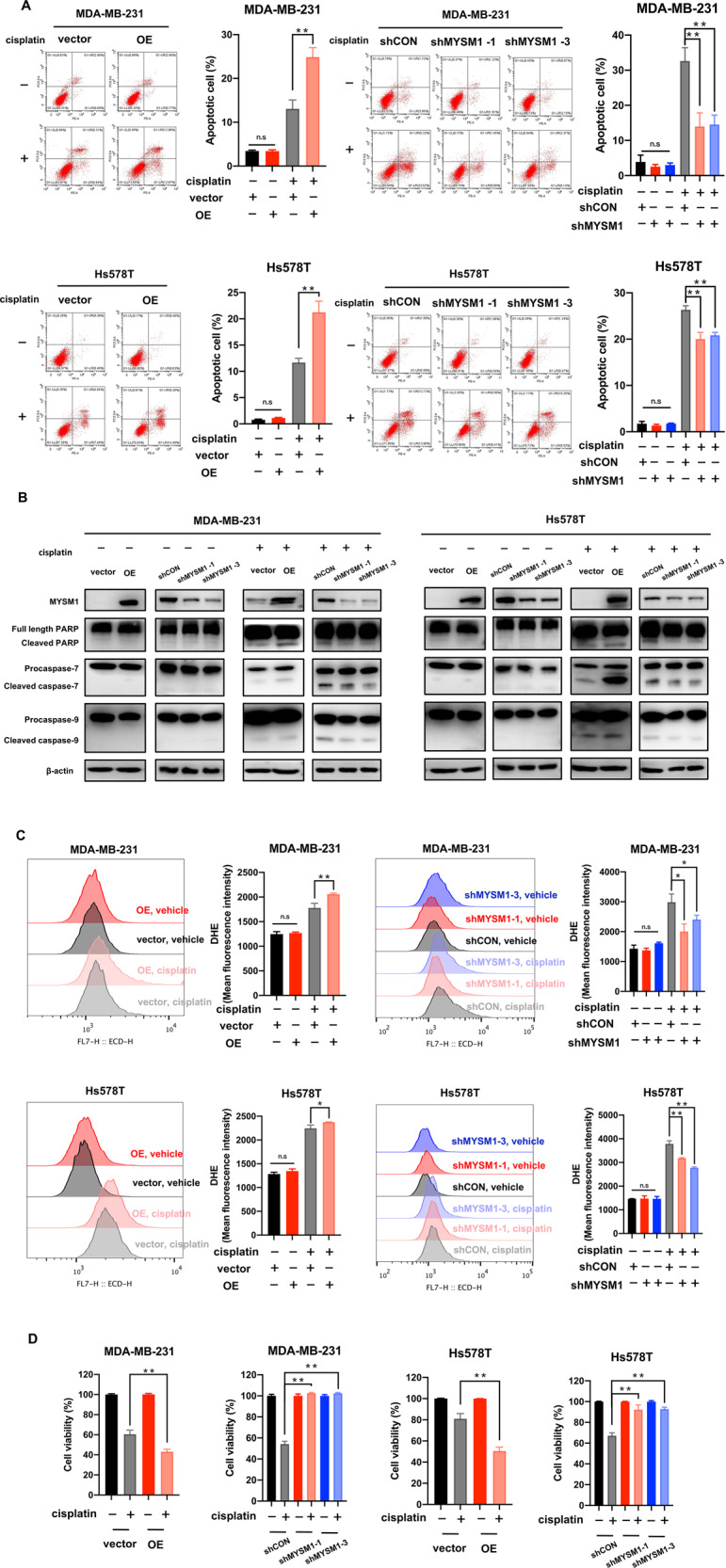
MYSM1 increases cellular apoptosis, ROS production, and decreases cell viability upon cisplatin treatment. MDA-MB-231 and Hs578T cells with MYSM1 overexpression and knockdown and their corresponding negative control cells were treated with or without cisplatin (5 µg/mL) for 48 h. **A** Flow cytometry was performed to detect PE/Annexin V staining and evaluate apoptosis. **B** The cleaved PARP, full-length PARP, cleaved caspase-7, procaspase-7, cleaved caspase-9, and procaspase-9 were detected by western blot. **C** Flow cytometry was performed to detect DHE staining and evaluate ROS production. **D** CCK8 assay was performed to measure cell viabilities. All data in the graph bars represent means ± SD. *P < 0.05 and **P < 0.01.

The apoptosis of cells overexpressing MYSM1 and gene knockdown after treatment with or without cisplatin (5 µg/mL) for 48 h was determined by flow cytometry to confirm whether MYSM1 mediated the cisplatin effect. The data showed no significant difference in the degree of apoptosis in cells not treated with cisplatin (Fig. 3A). The depleted MYSM1 remarkably reversed cisplatin-induced apoptosis in cisplatin-treated cells, whereas the increased expression of MYSM1 reinforced apoptosis (Fig. 3A).

The apoptosis marker cleaved PARP, cleaved caspase-7, cleaved caspase-9, and their corresponding full-length proteins [13] were detected by western blot. The cleaved PARP, cleaved caspase-7, and cleaved caspase-9 levels were increased in cells overexpressing MYSM1 upon cisplatin treatment. However, they were extremely low in the absence of cisplatin, which was consistent with the flow cytometry results (Fig. 3B). These findings suggested that MYSM1 exclusively mediated apoptosis in the presence of cisplatin.

ROS production, a marker of early apoptosis, was detected by flow cytometry. The data showed a similar trend under altered apoptosis. The ROS levels in cells remained unchanged without cisplatin treatment (Fig. 3C). In contrast, in the group of cells treated with cisplatin (5 µg/mL) for 48 h, the ROS levels were significantly higher in cells overexpressing MYSM1 and lower in cells with MYSM1 knockdown (Fig. 3C).

Furthermore, cell viability with or without cisplatin (5 µg/mL) treatment was determined via the CCK8 assay to confirm whether MYSM1 expression determined the cell’s fate upon cisplatin treatment. In line with the previous data, the upregulation of MYSM1 increased the cisplatin-induced cell death (Fig. 3D), indicating a higher sensitivity to cisplatin. Downregulation of MYSM1 reversed cell death. These findings suggested that MYSM1 might induce cisplatin sensitivity via cellular apoptosis.

### MYSM1 reduces cisplatin resistance via RSK3 inactivation and phospho-BAD (Ser 112) reduction

To elucidate the potential mechanism of MYSM1 affecting cisplatin resistance in TNBC cells, the empty vector-transfected and MYSM1 overexpressing MDA-MB-231 cells were incubated with cisplatin (5 µg/mL) for 48 h, the total RNA was extracted, and RNA sequencing was performed to analyze the transcriptomes (Supplementary Fig. 1). *RSK3*, a notable gene, showed significant changes. *RSK3* is a member of the p90 ribosomal S6 kinase family. RSKs are directly activated in transcriptional and cell cycle regulation via MEK/ERK signaling [14, 15]. RSK3 was previously reported to block BAD-mediated cell death by phosphorylating BAD at Ser 112 [16]. Because BAD is a crucial factor in apoptosis, MYSM1 was assumed to affect cellular apoptosis via the phosphorylation of BAD at Ser 112 by RSK3.

**Fig. 4:**
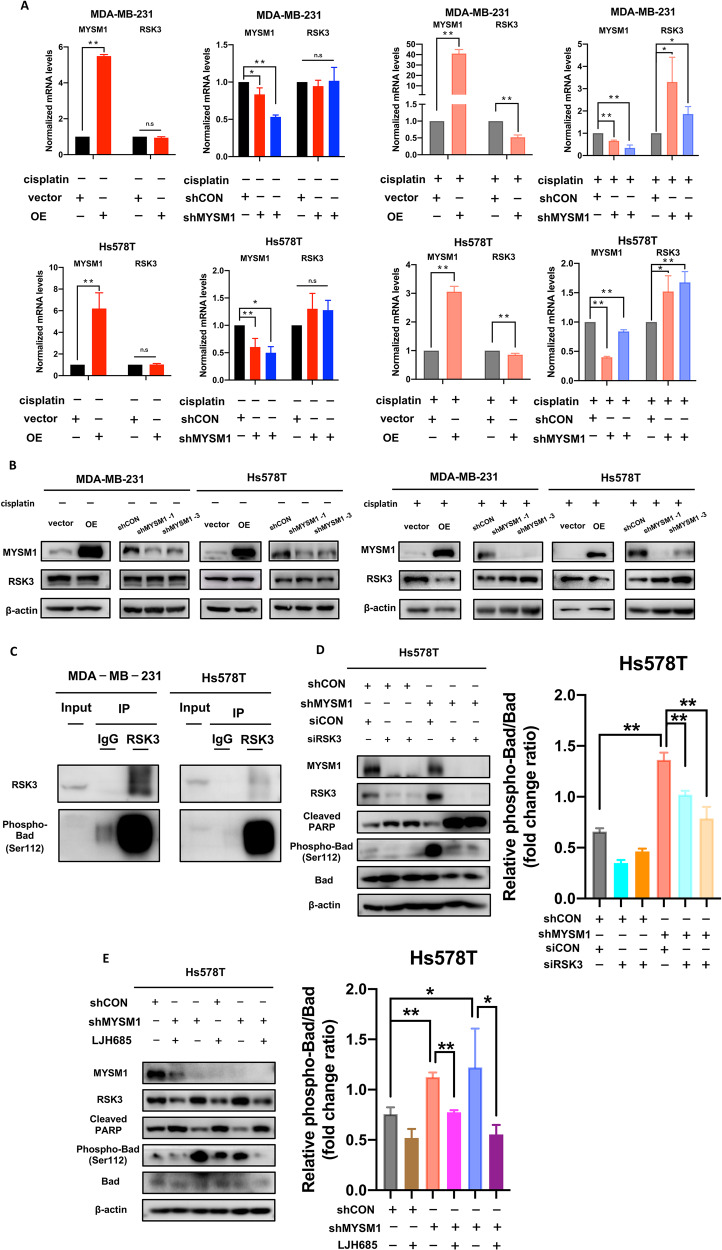
MYSM1 reduces cisplatin resistance by RSK3 inactivation and decreased phospho-BAD (Ser 112). **A** MYSM1 overexpressed and knockdown MDA-MB-231 and Hs578T cells and their corresponding negative control cells were treated with or without cisplatin (5 µg/mL) for 48 h. The whole-cell lysate was collected to extract mRNA, and quantitative real-time qPCR was performed to detect mRNA levels. **B** MYSM1 overexpressed and knockdown MDA-MB-231 and Hs578T cells and their corresponding negative control cells were treated with or without cisplatin (5 µg/mL) for 48 h. The whole-cell lysate was collected to extract protein, and western blot was performed to detect protein levels. **C** MDA-MB-231 and Hs578T cells were treated with cisplatin (5 µg/mL) for 48 h. RSK3 was immunoprecipitated with an RSK3-specific antibody. Endogenous RSK3 and phospho-BAD (Ser 112) were determined via western blot. **D**, **E** Following the corresponding treatments, cells were treated with cisplatin (5 µg/mL) for 48 h. Protein levels were detected by western blotting analysis. All data in the graph bars represent means ± SD. *P < 0.05 and **P < 0.01.

Consistent with the RNA-sequencing results, MYSM1 overexpression decreased the RSK3 mRNA (Fig. 4A) and protein levels (Fig. 4B) following cisplatin treatment. In contrast, the low expression of MYSM1 promoted RSK3 expression under cisplatin treatment. However, RSK3 expression did not change with MYSM1 expression in the absence of cisplatin treatment. The interaction between RSK3 and phospho-BAD (Ser 112) upon cisplatin treatment was investigated to validate whether RSK3 phosphorylated BAD at Ser 112. RSK3 was immunoprecipitated using an RSK3-specific antibody, and phospho-BAD (Ser 112) was detected via immunoblotting. Thus, an inherent interaction was found between RSK3 and phospho-BAD (Ser 112) (Fig. 4C) in cisplatin (5 µg/mL)-treated MDA-MB-231 and Hs578T parental cell lines. After cisplatin treatment, phospho-BAD (Ser 112)/BAD ratio level in shMYSM1 cells increased significantly following increased RSK3 level compared with that in shCON cells (Fig. 4D, E). Upon siRSK3 transfection (Fig. 4D) or LJH685 (RSK3 inhibitor) treatment (Fig. 4E) in shMYSM1 cells, phospho-BAD (Ser 112)/BAD ratio levels decreased, following a decreased RSK3 level, compared with siCON-transfected or vehicle-treated shMYSM1 cells. These results suggested MYSM1 regulated phospho-BAD (Ser 112) level by regulating RSK3 expression.

These findings proved that RSK3 phosphorylated BAD at Ser 112 upon cisplatin treatment and MYSM1 might control cisplatin resistance via the inactivation of RSK3 and subsequent reduction of phospho-BAD (Ser 112).

### RSK3, the downstream target of MYSM1, regulates cellular apoptosis and induces cisplatin resistance

The above results indicated that MYSM1 sensitized TNBC cells to cisplatin by increasing cellular apoptosis. Moreover, MYSM1’s downstream target, RSK3, could phosphorylate the pro-apoptotic factor, BAD, at Ser 112. The primary question was whether silencing RSK3 in shMYSM1 cells increased apoptosis and sensitivity to cisplatin.

**Fig. 5:**
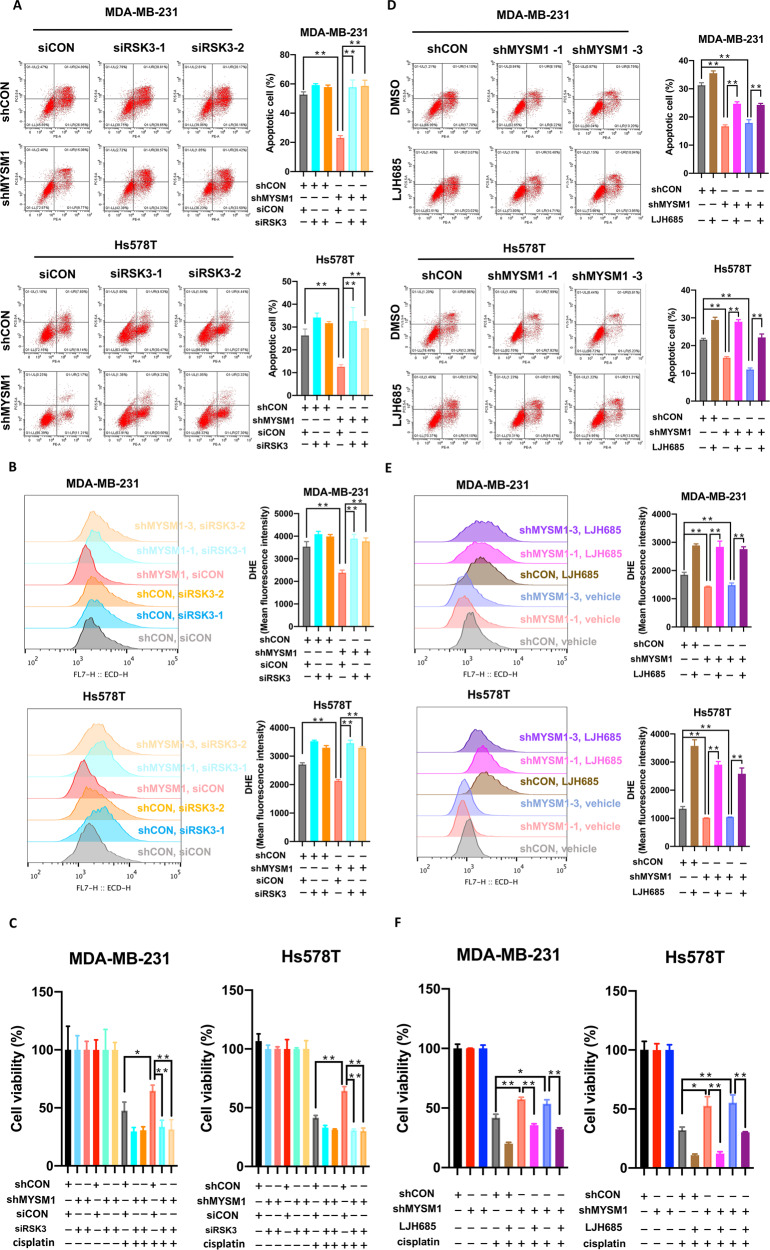
RSK3, the downstream of MYSM1, regulates cellular apoptosis and induces cisplatin resistance. The siRSK3s or negative control siRNA (siCON) were transfected into MYSM1 knockdown MDA-MB-231 and Hs578T cells, and their corresponding negative control cells. The cells were treated with or without cisplatin (5 µg/mL) for 48 h. **A** Flow cytometry was performed to detect PE/Annexin V staining and evaluate apoptosis. **B** Flow cytometry was performed to detect DHE staining to evaluate ROS production. **C** CCK8 assay was performed to measure the cell viability. The MYSM1 knockdown MDA-MB-231 and Hs578T cells and their corresponding negative controls were pretreated with 50 µM of LJH685 or DMSO for 24 h, followed by changing the medium to cisplatin (5 µg/mL) for 48 h. **D** Flow cytometry was performed to detect PE/Annexin V staining and evaluate apoptosis. **E** Flow cytometry was performed to detect DHE staining to evaluate ROS production. **F** CCK8 assay was performed to measure cell viability. All data in the graph bars represent means ± SD. *P < 0.05 and **P < 0.01.

siRSK3 or negative control siRNA (siCON) was transfected into MYSM1 knockdown (shMYSM1) cells and their corresponding control (shCON) cells. Then, the altered biological effects were monitored. Compared with shCON cells, lower cell apoptosis (Figs. 3A, 5A), lower ROS production (Figs. 3C, 5B), and higher cell viability (Figs. 3D, 5C) were observed in cisplatin-treated shMYSM1 cells. However, when RSK3 levels were lowered by siRSK3 transfection in shMYSM1 cells, cisplatin-induced cell apoptosis was restored and significantly rose up again (Fig. 5A). ROS production (Fig. 5B) and cell viability (Fig. 5C) changes were also reversed. Thus, the inhibition of the elevated RSK3 expression reversed the biological effects induced by MYSM1 reduction following cisplatin treatment, suggesting that RSK3 was the downstream target of MYSM1-regulating cisplatin sensitivity.

Similar results were observed in LJH685-treated cells (Fig. 5D–F). After being pretreated with LJH685 (50 µM) for 24 h, the shMYSM1 cells showed higher levels of apoptosis (Fig. 5D), ROS production (Fig. 5E), and lower cell viabilities (Fig. 5F) than the vehicle-treated shMYSM1 cells. This result offered strong evidence that MYSM1 mediated cellular apoptosis and TNBC cells’ sensitivity to cisplatin by regulating the RSK3 expression.

These findings suggested that RSK3, which is the downstream target of MYSM1, altered cellular apoptosis and finally controlled cisplatin resistance in tumors.

## Discussion

MYSM1 is an H2A-specific DUB. It catalyzes the ubiquitin of monoubiquitinated histone H2A at Lysine 119. Previous studies of MYSM1 focused on hematopoietic and immune systems [17–19]. MYSM1 has been reported to regulate the differentiation and development of natural killer cells [17], along with the maintenance, self-renewal, and differentiation of hematopoietic stem cells (HSCs) [18] and bone marrow mesenchymal stem cells (BMSCs) [19]. Studies focusing on cancer demonstrated that MYSM1 was involved in cancer cell proliferation, cell cycle, migration, and invasion in colon cancer, pancreatic cancer, and melanoma [6–8]. The function of MYSM1 in breast cancer has yet to be reported. Here, this study reported that MYSM1 suppressed proliferation in TNBC and depleted cell resistance to cisplatin by promoting cellular apoptosis via the RSK3–phospho-BAD pathway.

MYSM1 was downregulated in CRPC, knocking down MYSM1 promoted proliferation and suppressed cell senescence in vivo [6]. Silencing MYSM1 in melanoma significantly reduced survival and proliferation [8]. The expression of MYSM1 was found to be reduced in breast cancer and correlated with a potentially worse prognosis (Fig. 1). On account of the worst prognosis of TNBC, the involvement of MYSM1 in TNBC aroused the interest of this study’s authors. Investigating the biological effects of MYSM1 overexpression and silencing TNBC cells suggested that MYSM1 might play an anticancer role by inhibiting cell proliferation in TNBC (Fig. 2). The previous studies involving CRPC [6] and melanoma [8] reported a similar biological function of MYSM1 as in TNBC, indicating that MYSM1 might act similarly in different types of cancer. Interestingly, a synergistic effect between MYSM1 and cisplatin treatment (Fig. 2C) hinted that MYSM1 might be involved in TNBC cisplatin treatment.

Cisplatin is widely used in anticancer chemotherapy in many kinds of cancers, including TNBC. Cisplatin plays an anticancer role mainly via the formation of Pt-DNA complex, causing DNA damage, cell cycle arrest, and apoptosis [20]. MYSM1 was involved in DNA damage caused by chemotherapy [21]. Moreover, MYSM1 repressed the p53-target gene *Bbc3*/*PUMA* to regulate hematopoietic progenitor cells’ apoptosis, ROS production, and DNA damage level [22]. Because cisplatin induces apoptosis, these findings prompted whether MYSM1 regulated apoptosis in cisplatin-treated TNBC. Cells did not display altered apoptosis in the absence of cisplatin treatment (Fig. 3A–B), showing a similar result to a previous report in hematopoietic progenitor cells [22]. Following cisplatin treatment, MYSM1 enhanced cisplatin-induced apoptosis and increased sensitivity to cisplatin (Fig. 3). However, after irradiation, MYSM1 silencing HSCs tended to be of higher cellular apoptosis and cell death percentages [22]. These results might reveal different mechanisms between tumor cells and HSCs, necessitating more research. Overall, MYSM1 was found to sensitize TNBC cells to cisplatin by opening the cellular switch that regulated apoptosis.

This study identified RSK3 as an important downstream target of MYSM1 upon cisplatin treatment in the RNA-sequencing result analysis. *RSK3* is a member of the RSK family. The RSK family, containing RSK1–4, are directly regulated via the ERK signaling pathway and are involved in cell cycle, proliferation, cell migration, and cell survival in cancer [14, 23–27]. Notably, previous studies have reported that RSK2 depletion reversed cell survival by inhibiting pro-apoptotic BAD [16, 28, 29] and BimEL [30], and thus increased the sensitivity of ovarian cells to apoptotic stimuli such as cisplatin [31]. Depleted RSK3 levels were observed in MYSM1-induced cisplatin-sensitive TNBC cells in this study. Furthermore, BAD was phosphorylated by RSK3 [16] and RSK1 [32] at Ser 112. An endogenic interaction was also found between RSK3 and phospho-BAD (Ser 112) in the presence of cisplatin (Fig. 4C). The inhibition of the increased RSK3 levels in shMYSM1 TNBC cells by siRSK3 transfection (Fig. 4D) or LJH685 treatment (Fig. 4E) drastically reduced the radio of phospho-BAD (Ser 112)/BAD, suggesting that the RSK3 expression regulated phospho-BAD (Ser 112) levels in the presence of cisplatin. A previous study showed that RSK3 phosphorylated BAD at Ser 112 and then mediated 14-3-3 proteins binding to reduce the pro-apoptotic function of BAD and the following PARP family, and finally reversed cellular apoptosis, leading to cell survival [16]. Apoptosis, ROS production, and cell viability changes could also be reversed by reducing RSK3 levels (Fig. 5). Therefore, this result suggested that MYSM1 sensitized TNBC cells to cisplatin by changing RSK3 and phospho-BAD (Ser 112) levels, causing the unbalance between cell apoptosis and survival.

**Fig. 6:**
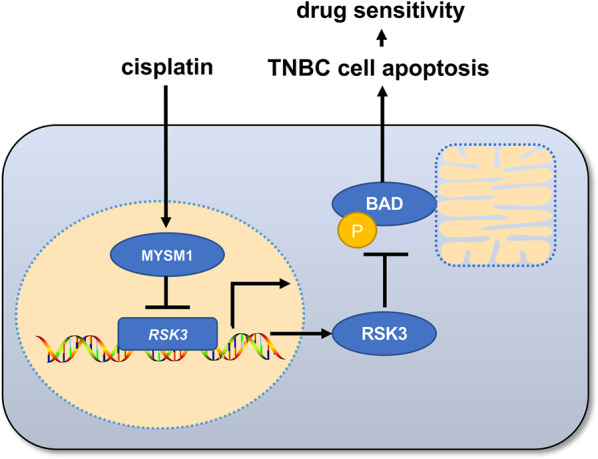
A schematic representation of MYSM1-mediated RSK3 expression and cisplatin resistance. Upon cisplatin treatment on TNBC cells, MYSM1 represses RSK3 expression, and reduces the phosphorylation of BAD at Ser 112, leading to cell apoptosis and cisplatin sensitivity.

In summary, this study’s data supported the first formal report that MYSM1 decreased RSK3 levels, resulting in the declined phosphorylation of BAD (Ser 112) as well as elevated cellular apoptosis and sensitivity to cisplatin in TNBC (Fig. 6). This finding suggests that MYSM1 plays a crucial role in TNBC and represents a potential new therapeutic target for chemotherapy in TNBC.

## Materials and methods

### Immunohistochemical analysis

Ten pairs of tissue samples comprising TNBC para-carcinoma and carcinoma tissues were collected from surgical patients diagnosed with TNBC at the Department of Breast Surgery, Fudan University, Shanghai Cancer Center (Shanghai, China). All patients gave their informed consent for participation. The diagnosis and pathological reports were clear.

The paraffin-embedded tissue sections were stained with the corresponding antibodies. In brief, the slides were deparaffinized, rehydrated, and treated with 3% H_2_O_2_. After that, the slides were boiled with sodium citrate buffer (10 mM, pH 6.0). After blocking with 3% fetal bovine serum (FBS), the slides were incubated with primary antibodies (MYSM1, 20078-1-AP, 1:800 dilution, Proteintech, USA) at 4 °C overnight. Then, the slides were incubated with secondary antibodies (D-3004, Long Island Antibody, China). Finally, the specimens were stained with 3,3′-diaminobenzidine and hematoxylin. Results were obtained with Image-Pro Plus software program (Media Cybernetics). The staining intensity was scored as follows: 1, no staining; 2, weak staining; 3, intermediate staining; and 4, dark staining. The MYSM1 expression was scored as a product of positive cell percentage and staining intensity score.

### Cell lines and cells culture

The human breast cancer MDA-MB-231 and Hs578T cell lines were obtained from the Department of Breast Surgery, Fudan University, Shanghai Cancer Center (Shanghai, China). MDA-MB-231 and Hs578T cells were cultured in low-glucose Dulbecco’s modified eagle medium supplemented with 10% FBS and 1% penicillin–streptomycin. Cells were incubated at 37 °C with 5% CO_2_ in a humidified environment.

### Lentivirus infection and transient transfection

Cell lines with stable MYSM1 overexpression (vector cells and overexpressing cells) and knockdown (shCON cells and shMYSM1 cells) were established by lentivirus infection. The lentivirus was purchased from Genechem (China). MDA-MB-231 and Hs578T cells were seeded at a density of 1 × 10^5^ per well in six-well plates and infected with lentivirus. Following overnight infection, the medium was replaced with 2 µg/mL of puromycin and incubated for at least three days until the uninfected cells died. Supplementary Table 1 enlists the shRNA sequences of this study.

For transient transfection, MDA-MB-231 and Hs578T cells in six-well plates were infected with 5 µM of siRNA (Sangon Biotech, China) and 5 µL of RNATransMate (E607402, Sangon Biotech, China). The cells were used in subsequent experiments. Supplementary Table 1 shows the siRNA sequences used in this study.

### Protein extraction and western blot

Cells were harvested after treatment, and the whole-cell protein was extracted with western IP lysis buffer (P0013J, Beyotime, China) supplemented with protease inhibitors. Protein concentrations were measured using a BCA Protein Quantification Kit (20201ES76, YEASEN, China). Protein samples were separated electrophoretically and transferred to polyvinylidene fluoride membranes (ISEQ00010, Merck Millipore, USA) and blocked with 2% non-fat milk (A600669, Sangon Biotech, China). The primary antibodies consisted of β-actin (66009-1-Ig, 1:3000, Proteintech, USA), MYSM1 (ab193081, 1:1000, Abcam, UK), RSK3 (DF8603, 1:500, Affinity, China), cleaved PARP (5626, 1:500, Cell Signaling Technology, USA), PARP (9532, 1:1000, Cell Signaling Technology, USA), Caspase-7 (12827, 1:1000, Cell Signaling Technology, USA), Caspase-9 (9508, 1:1000, Cell Signaling Technology, USA), and phospho-BAD (Ser 112) (5284, 1:500, Cell Signaling Technology, USA), BAD (9239, 1:500, Cell Signaling Technology, USA). The secondary antibodies comprised HRP-conjugated Affinipure Goat Anti-Rabbit IgG (SA00001-2, 1:3000, Proteintech, USA) and HRP-conjugated Affinipure Goat Anti-Mouse IgG (SA00001-1, 1:3000, Proteintech, USA).

### RNA extraction and quantitative real-time PCR

RNA was isolated using an RNA-Quick Purification Kit (RN001, ES Science, China) and reverse-transcribed using a PrimeScript RT Master Mix (Perfect Real Time) (RR036A, TAKARA, Japan) according to the manufacturer’s protocols. The quantitative real-time PCR was performed using the 20 µL of reaction system containing 200 ng cDNA, 0.25 µM of primers (Supplementary Table 2), 10 µL of Hieff UNICON® Universal Blue qPCR SYBR Green Master (11184ES03, Yeasen, China), and ddH_2_O. The reaction program was performed at 95 °C for 2 min, 40 cycles of 95 °C for 10 s and 60 °C for 30 s using a Mastercycler® ep realplex (QuantStudio^TM^ DX, Thermo Fisher Scientific, USA). The relative expression levels of mRNA were calculated using the 2^−ΔΔCt^ method. Supplementary Table 2 enlists the primers sequences of this study.

### Colony formation assay

One thousand cells were seeded into each well in the 6-well plates and incubated for 10–14 days until visible colonies of at least 50–100 cells appeared. Colonies were counted after being stained with 0.5% crystal violet.

### EdU assay

The EdU kit (C10310-2, Cell-LightTM EdU Apollo488 In Vitro Kit) was purchased from RIBOBIO (China). MDA-MB-231 (3 × 10^4^ cells) or Hs578T (5 × 10^4^ cells) were plated in 24-well plates, adhered overnight, and subjected to the corresponding treatment. Cells were incubated with 50 µM of EdU solution for 2 h at 37 °C and fixed with 4% paraformaldehyde. The reaction was terminated with 2 mg/mL of glycine. Cells were treated with 0.5% Triton X-100. The 1× Apollo staining solution was added and incubated in the dark for 30 min, followed by washing with 0.5% Triton X-100. Finally, the images were visualized and acquired with Leica DMI6000B/DFC365FX (German). All operations were conducted according to the manufacturer’s instructions.

### Apoptosis assay

MDA-MB-231 and Hs578T cells were seeded at a density of 2 × 10^5^ per well in six-well plates and treated accordingly. Cells were harvested and washed twice with PBS. Apoptosis was detected by staining the cells with Annexin V and 7-AAD according to the instructions indicated in the PE Annexin V Apoptosis Detection Kit I (559763, BD Biosciences, USA). Stained cells were analyzed using CytoFLEX S (Beckman Coulter, USA).

### ROS assay

MDA-MB-231 and Hs578T cells were seeded at a density of 2 × 10^5^ per well in six-well plates and treated appropriately. The ROS analysis was performed by diluting dihydroethidium (S0063, Beyotime, China) with a pre-heated complete medium to 5 μM and incubating cells at 37 °C in the dark for 20 min. Stained cells were analyzed and quantified using CytoFLEX S (Beckman Coulter, USA).

### Cell viability

MDA-MB-231 and Hs578T cells were seeded at a density of 3 × 10^3^ per well in 96-well plates and cultured for 24 h, followed by the corresponding treatment. The assay was performed at the indicated time points according to instructions provided in the Cell Counting kit-8 (40203ES60, Yeasen, China). The plates were incubated at 37 °C for 2 h, and the optical density (OD) was read at 450 nm with a microplate reader (Spectramax M5, Molecular Devices, USA). The cell viability of one certain cell was equal to the OD of this certain cell treated with or without drug, divided by the OD of this certain cell treated without drug.

### RNA sequencing

The empty vector-transfected and MYSM1 overexpressing MDA-MB-231 cells were treated with cisplatin (P4394, Sigma-Aldrich, USA) (5 µg/mL) for 48 h. The total RNA was extracted using TRIzol. RNA sequencing and data analysis were performed by GENEWIZ (China).

### Co-immunoprecipitation

Cells were scraped following treatment and the whole-cell protein was extracted with NP40 lysis buffer (P0013F, Beyotime, China) supplemented with protease inhibitors. The whole-cell protein was incubated with the RSK3 polyclonal antibody (14446-1-AP, Proteintech, USA) or the Normal Rabbit IgG (2729, Cell Signaling Technology, USA) overnight at 4 °C, then incubated with protein A/G agarose beads (sc-2003, Santa Cruz, USA) for 2 h, at 4 °C. The beads were washed five times with NP40 lysis buffer. The beads were boiled before performing the western blot.

### Statistical analysis

Prism 8 software was used to perform statistical analysis. Wilcoxon test, ANOVA, and Student’s *t* test were used. *P* < 0.05 was considered statistically significant for this study.

## Supplementary information

Supplementary Figure 1-Volcanic map of RNA-seq differential genes analysis

Supplementary Table 1

Supplementary Table 2

## Data Availability

The data used and analyzed in this study are available from the corresponding authors on reasonable request.
