# Supplementary material for: MYSM1 induces apoptosis and sensitizes TNBC cells to cisplatin via RSK3–phospho-BAD pathway
Source: Cell Death Discov. 2022 Feb 26;8:84. doi: 10.1038/s41420-022-00881-1 (PMC8881619; doi:10.1038/s41420-022-00881-1)
Supplement: Supplementary file 1 — Supplementary Figure 1-Volcanic map of RNA-seq differential genes analysis [file 41420_2022_881_MOESM1_ESM.docx]

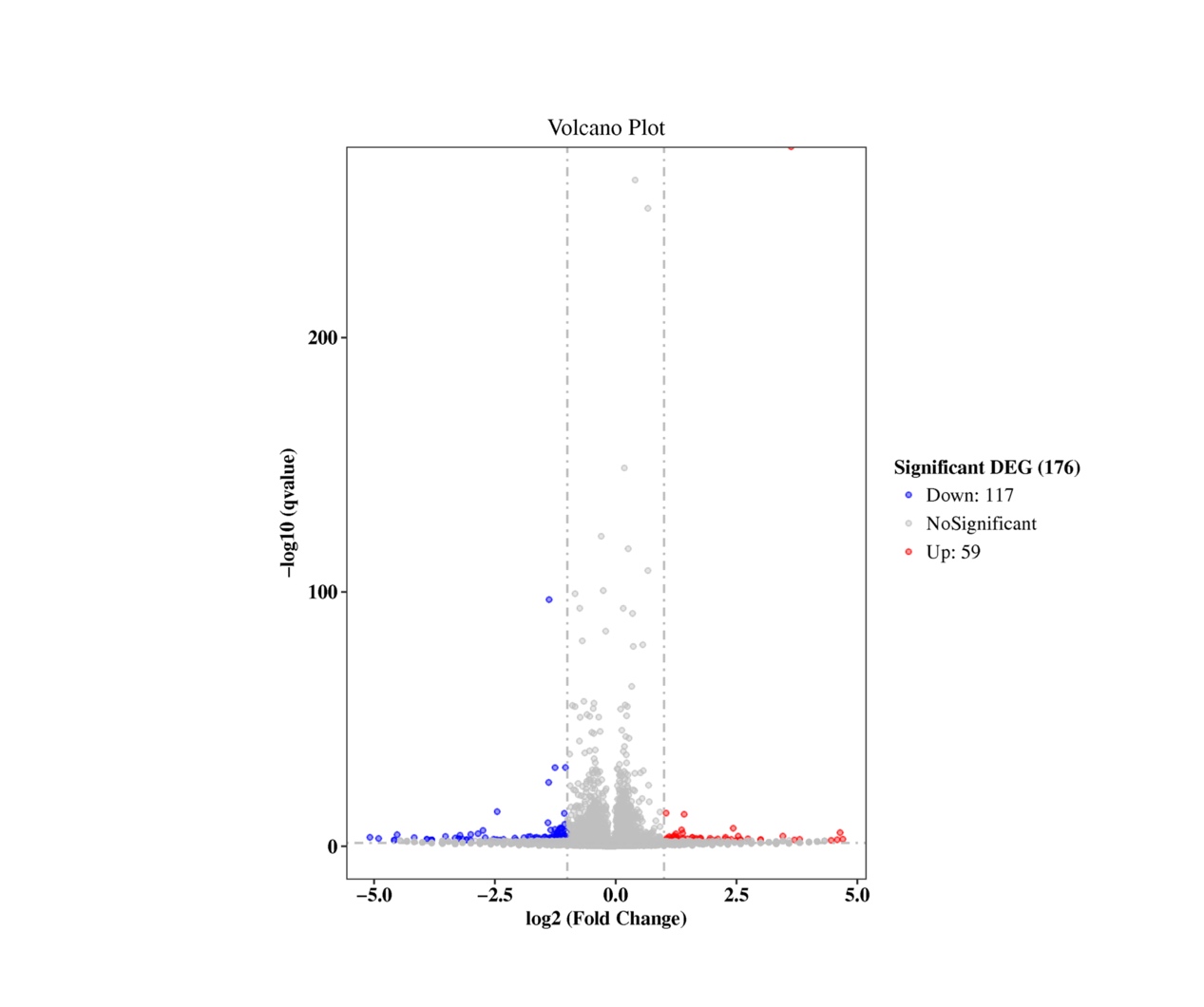


**Figure Legends**

Supplementary Figure 1. Volcanic map of RNA-seq differential genes analysis

The empty vector transfected and MYSM1-overexpressing MDA-MB-231 cells were treated with cisplatin (5 µg/mL) for 48 h. RNA sequencing and data analysis were performed. *P* <0.05, |log2FC| $\geq$1 were set to detect differential expressed genes.
